# Immunogenic cell death in cancer therapy: Present and emerging inducers

**DOI:** 10.1111/jcmm.14356

**Published:** 2019-06-18

**Authors:** Jingyi Zhou, Gangyang Wang, Yinze Chen, Hongxia Wang, Yingqi Hua, Zhengdong Cai

**Affiliations:** ^1^ Department of Orthopaedics Shanghai Bone Tumor Institute, Shanghai General Hospital, Shanghai Jiao Tong University School of Medicine Shanghai China; ^2^ Fourth Clinical College Xinxiang Medical University Henan China; ^3^ Department of Oncology Shanghai General Hospital, Shanghai Jiao Tong University School of Medicine Shanghai PR China

**Keywords:** antitumour effects, damage‐associated molecular patterns, dendritic cells, ICD inducers, immune system, immunogenic cancer cell death

## Abstract

In the tumour microenvironment (TME), immunogenic cell death (ICD) plays a major role in stimulating the dysfunctional antitumour immune system. Chronic exposure of damage‐associated molecular patterns (DAMPs) attracts receptors and ligands on dendritic cells (DCs) and activates immature DCs to transition to a mature phenotype, which promotes the processing of phagocytic cargo in DCs and accelerates the engulfment of antigenic components by DCs. Consequently, via antigen presentation, DCs stimulate specific T cell responses that kill more cancer cells. The induction of ICD eventually results in long‐lasting protective antitumour immunity. Through the exploration of ICD inducers, recent studies have shown that there are many novel modalities with the ability to induce immunogenic cancer cell death. In this review, we mainly discussed and summarized the emerging methods for inducing immunogenic cancer cell death. Concepts and molecular mechanisms relevant to antitumour effects of ICD are also briefly discussed.

**Main topics**

- Immunogenic cell death (ICD) is defined by chronic exposure of damage‐associated molecular patterns (DAMPs) in the tumour microenvironment (TME), which stimulates the dysfunctional antitumour immune system.
- The induction of ICD contributes to long‐lasting protective antitumour immunity.
- ICD induction via physical therapy and combination therapy has emerged as novel cancer therapies.

## 1 INTRODUCTION

During the multistep progression of cancer, immune surveillance, an immune process that recognizes and destroys numerous derailed cells,^1^ is regarded as a regulator in the context of normal cell differentiation, cancer cell proliferation and cell death mechanisms. To escape immune surveillance, malignant cells develop different strategies to subjugate the immune system and create an environment that supports their proliferation. If the functionality of the immune system is reduced for a period of time, cancer incidence and recurrence rates may increase. Therefore, thanks to the organism's positive mechanisms of the activated immune system and enhanced immune surveillance, aberrant cells remain completely latent.^2^

Determining the impacts of the immune system on cancer cells is important for the development of cancer treatments. Currently, there are two main strategies for eliciting antitumour effects via the immune system. First, immune system components, such as antibodies, natural killer (NK) cells or other immune cells, including T cells, which are born to effectively instruct passive immunity, can be exploited as ‘killing’ factors. After being activated by interleukin‐2(IL‐2), NK cells can hunt and kill tumour cells directly, without a requirement for prior sensitization for efficient tumour cell lysis.^3^ In contrast, antibodies, from B cells or injections, bind to antigens or receptors on the surface of cancer cells, destroying protumour cytokines and automatically blocking the interaction between cancer cells and the microenvironment.^4^ Second, to establish active immunity, antigen presenting cells (APCs), such as dendritic cells (DCs), function as pivotal regulators of immune outcome, such as tolerance or immune activation.^5^ After loading with tumour‐associated antigen and proper antigen processing, DCs produce pro‐inflammatory cytokines, which activate tumour‐specific cytotoxic T lymphocytes (CTLs) to induce immune‐mediated killing.^6^ However, as the sentinel APCs of the immune system, the maturation status of DCs determines the efficiency and ultimate success of their interaction with cancer cells because fully mature DCs can provide all three conventional T cell stimulatory signals, enabling the elicitation of potent anticancer immunity; this kind of productive interface with dying cells is regarded as a necessary form of communication.^7^ Although killing cancer cells is the most direct method of immune clearance, it has recently been found that prior to pathogen reproduction and transmission during an infection, the first batch of pathogen‐infected cells actively commits suicide; then, the dead cell debris is quickly cleared by macrophages or neighbouring cells without affecting the normal functions of other cells. We have confirmed that this non‐inflammatory cell death is programmed cell death (PCD).

**Figure 1:**
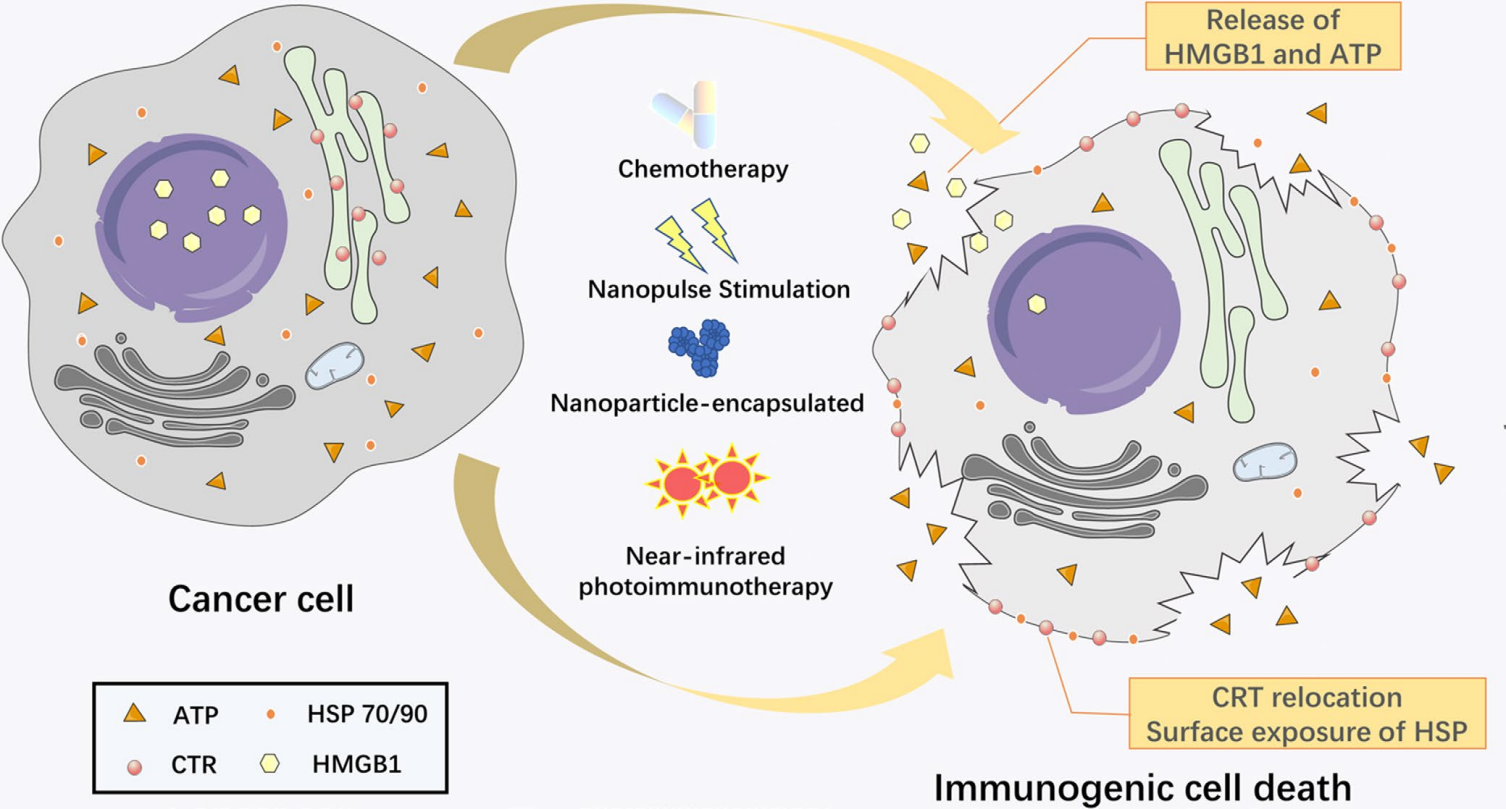
Schematic representation of the induction of immunogenic cell death (ICD). After treatment with different ICD inducers, cancer cells are induced to undergo apoptosis, which leads to cell swelling and bursting. Subsequently, the dying cells express damage‐associated molecular pattern (DAMPs) hallmarks, including the translocation of CRT from the endoplasmic reticulum to the cell surface, the release of high mobility group B1 from the nucleus, the extracellular secretion of adenosine triphosphate and the expression of HSPs on the cell surface

PCD, or more specifically, apoptosis, is a unique strategy for protecting a host from every possible pathogen.^8^ Through the activation of caspase‐3, both the intrinsic and extrinsic pathways are activated and more than 500 cellular substrates are cleaved to execute the apoptosis program. The ‘intrinsic’ apoptotic pathway, is regulated by pro‐apoptotic members of the BCL‐2 family, which stimulates mitochondria to release molecules such as cytochrome c,^9^ which together with pro‐caspase‐9 and apoptotic protease‐activating factor 1 (APAF1), forms the apoptosome, a multiprotein complex induct PCD.^10^, ^11^ In contrast, the ‘death receptor’ pathway, is carried out by the formation of a multiprotein complex containing FAS, adaptor protein FAS‐associated death domain (FADD)^12^ and pro‐caspase‐8, which is known as the death‐inducing signalling complex (DISC).^13^ Normally, apoptotic cells are rapidly engulfed by macrophages, but unlike the swelling and rupturing that occur during the necrosis process, which are truly immunogenic, apoptotic cell death has long been hypothesized to be poorly immunogenic (or even tolerogenic).^14^ However, some dying apoptotic cells release their cellular contents and these contents contain damage‐associated molecular patterns (DAMPs), which act as danger signals to produce immunostimulatory effects, including the recruitment and activation of neutrophils, macrophages and other immune cells.^8^ Moreover, the apoptotic nature of cell death is critical for determining immunogenicity.^15^ Immunogenic cell death (ICD) is defined by the chronic exposure of DAMPs to the immune system, which may drive autoimmunity and promote immune‐mediated elimination in the tumour microenvironment (TME) (Figure 1).

The induction of ICD is regarded as stressor dependent because endoplasmic reticulum (ER) stress and reactive oxygen species (ROS) production are the required components for the exposure of different DAMPs. The ER is a eukaryotic organelle that responds to stress by activating a complex signalling pathway, ER stress is henceforth a general ‘enabler’ of ICD, known as disturbed ER homeostasis.^16^ When combined with ROS production, the activated danger signalling pathway helps traffic DAMPs to the extracellular space, where they are presented at the cell surface and function as ‘eat me’ signals for recruited immune cells.^17^, ^18^ However, last year, Giampazolias et al found a novel pathway that induces a new type of ICD, which kills cells by mitochondrial outer membrane permeabilization (MOMP), even without caspase activity. They named this phenomenon MOMP‐induced caspase‐independent cell death (CICD). Furthermore, MOMP can stimulate NF‐κB activity during CICD through down‐regulating the expression of inhibitor of proteins that apoptosis, leading to NF‐κB‐inducing kinase (NIK) stabilization and activation, which triggers cell death that is classified as ICD.^19^ Therefore, the association between the ER and MOMP‐CICD is unknown, but the pathway that induces ICD has not been fully explored.

Accordingly, in this review, we discuss the molecular mechanisms of ICD in the context of cancer treatment and in view of the therapeutic effect of ICD, we focus on reviewing the emerging methods for inducing immunogenic cancer cell death, as well as clinical studies of novel ICD inducers and potential applications in human oncology.

## 2 ICD IN CANCER THERAPY

ICD provides a new opportunity to improve the effectiveness of cancer treatment and relieve the suffering of patients. ICD involves the killing of cells induced not only by ICD inducers but also by dying cancer cells, which act as a tumour vaccine, causing a tumour‐specific immune response that targets live cancer cells and residual tumour tissue. In this way, patients can obtain long‐term clinical benefits from a treatment response initiated by cytotoxic chemotherapy and physical induction.^20^

**Figure 2:**
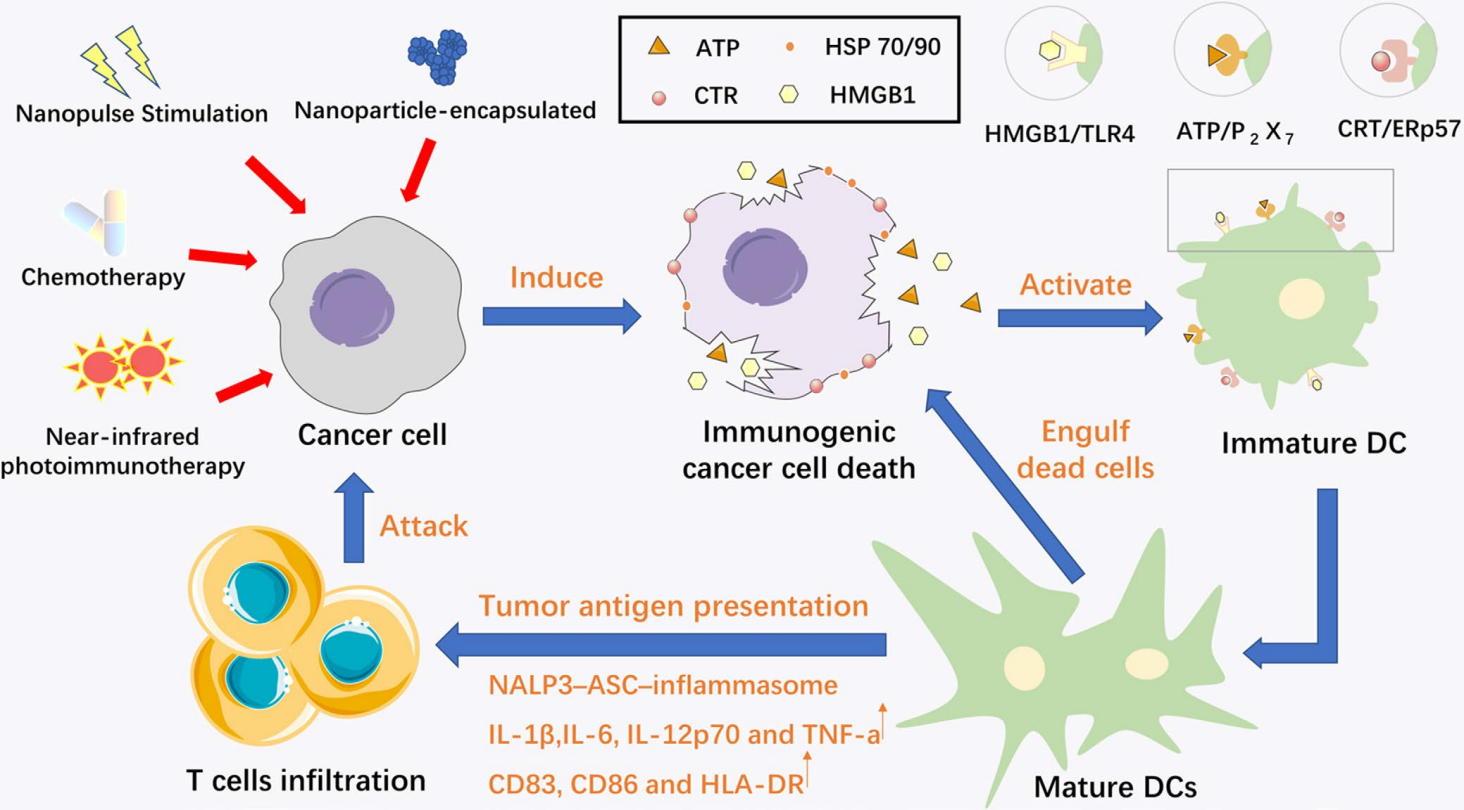
A schematic explaining the mechanism by which immunogenic cell death (ICD) is induced in dendritic cells and the effects of this progress on host immunity. After the induction of ICD, chronic exposure of damage‐associated molecular patterns (DAMPs) on cancer cells attracts receptors and ligands on dendritic cells (DCs) and activates immature DCs to transition to a mature phenotype. CRT/ERp57 provides an ‘eat me’ signal that promotes phagocytosis of the cell by DCs; similarly, extracellularly secreted adenosine triphosphate is regarded as a ‘find me’ signal, which triggers P_2_X_7_ receptors on DCs and is responsible for the activation of the NALP3‐ASC‐inflammasome and the secretion of IL‐1β. The binding of high mobility group B1 to Toll‐like receptor 4 (TLR4) and the expression of HSP70/90 have immunostimulatory properties that promote the processing of phagocytic cargo in DCs and accelerate the engulfment of antigenic components by DCs, which consequently stimulate specific T cell responses and the killing of more cancer cells

During the cell death process of ICD, immunogenic dead cells expose different hallmarks on the cell surface and release different substances to interact with APCs or other immune cells. These molecules that mediate immunogenicity are considered to be DAMPs, which are responsible for the ‘anticancer vaccine effect’ of ICD^21^ (Figure 2). In the pre‐apoptotic stage, immunogenic dead cells translocate calreticulin (CRT, a 46 kDa Ca^2+^‐binding protein), from the perinuclear ER to the cellular periphery and similarly relocalize ERp57.^22^ Once the CRT/ERp57 complex is exposed on the cell surface, it provides an ‘eat me’ signal to promote phagocytosis by DCs.^15^, ^23^ Moreover, the exposure of CRT on the surface of cancer cells succumbing to ICD also induces tumour antigen presentation and tumour‐specific CTL responses.^15^

During the blebbing phase of apoptosis, another hallmark of ICD is the secretion of adenosine triphosphate (ATP) from dying cancer cells, which is regarded as a ‘find me’ signal. However, autophagy is required for the secretion of ATP from dying cancer cells. Autophagy is regarded as a pre‐mortem stress adaptation mechanism that degrades cytoplasmic proteins, aggregates and damaged organelles through a catabolic process. During its degradation, the autophagosome‐lysosome complex and the fusion of lysosomal and plasma membranes eventually allow ATP exocytosis and constitute the main source of extracellular ATP release from the intracellular environment.^24^, ^25^ Furthermore, the release of ATP acts as a chemoattractant for DC precursors^26^ and activates purinergic P2X7 receptors on DCs, triggering the NALP3‐ASC‐inflammasome and driving the secretion of IL‐1β.^27^ Through this pathway, important cytokines are provided in the context of antigen presentation, which is also required for the adaptive immune response to cancer cells that is mediated by the polarization of interferon‐γ (IFNγ)‐producing CD8^+^ T cells.^17^

High mobility group B1 (HMGB1) is a non‐histone chromatin‐binding protein. In the late stages of apoptosis, cells are damaged and disrupted and HMGB1 is released from the nucleus, which can be detected by an enzyme‐linked immunosorbent assay (ELISA); accumulation of extracellular HMGB1 also occurs at this stage.^22^, ^28^, ^29^ It has been widely reported that the binding of HMGB1 (released from immunogenic dying cells) to Toll‐like receptor 4 (TLR4, mainly expressed on DCs) is vital for activating dendritic cells and facilitating antigen presentation by DCs to T cells. Moreover, the recognition of HMGB1 by TLR4 subsequently triggers MyD88 (the primary myeloid differentiation response gene), the adapter for TLR4. The TLR4/ MyD88 pathway enhances tumour antigen processing by inhibiting fusion between phagosomes and lysosomes, which promotes the processing of phagocytic cargo in DCs and accelerates the engulfment of antigenic components by DCs.^30^, ^31^ As one of the characteristics of ICD, the expression of ecto‐HSP70 and ecto‐HSP90 on dying cell membranes has immunostimulatory properties, which lead to specific CD8^+^ T cell responses by driving the cross‐presentation of tumour‐derived antigenic peptides on major histocompatibility complex (MHC) class I molecules.^32^, ^33^ Accordingly, CRT exposure, ATP secretion and HMGB1 release by human cancer cells appear to be the gold‐standard for accurately predicting the ICD‐inducing capacity of chemotherapeutic agents.

Interactions between these DAMPs and phagocytosis receptors, purinergic receptors and pattern‐recognition receptors (PRRs) on the surface of innate immune cells, which act as activators that stimulate APCs to present antigens on MHC I and MHC II molecules to T cells and trigger T cell immune response against cancer‐specific antigens, subsequently elicit protective anticancer immune responses in vivo. The interface of ICD with DCs through DAMPs has been regarded as a pivotal process that turns cancer cells into tumour vaccines and mediates immune clearing of all cancer cells, which makes ICD unique and incredibly beneficial for cancer therapy. Furthermore, the inflammatory response and neutrophilic inflammation are ‘additional’ effects that are induced by ICD.^34^ Abhishek et al^35^ revealed that via pathogen‐associated molecular patterns(PAMP)‐triggered activation, immunogenic dying cancer cells could trigger pathogen response‐like chemokine (PARC) signatures, with co‐release of the chemokines CXCL1, CCL2 and CXCL10. Both GO bioinformatics analysis and unbiased correlation analysis indicated that under the influence of PAMPs influence, the recruitment of neutrophils was maximally positively correlated with the release of CXCL1, CCL2 and CXCL10. In other words, the special neutrophil‐attracting phenotype of ICD could recruit neutrophils as the first innate immune responders, triggering neutrophil‐driven phagocytosis and pro‐inflammatory stimulation. However, other researchers further explored this issue and revealed that the secretion of CXCL 1 leads to neutrophilic inflammation in a MyD88‐ and IL1R‐dependent manners.^36^ Consequently, immunogenic dying cells could recruit neutrophils for phagocytosis of dead/dying cancer cells and cytotoxic targeting of residual cancer cells.

The above discussion indicates that it is essential to understand the emerging methods of ICD induction and apply them to clinical cancer treatments.

## 3 EMERGING METHODS OF ICD INDUCTION

Since the unique cancer cell killing function of ICD was confirmed, ICD inducers have been a popular research focus. Some classic ICD inducers, such as anthracyclines (doxorubicin, idarubicin and mitoxantrone),^37^ all the chemical PP1/GADD34 inhibitors (tautomycin, calyculin A and salubrinal),^15^, ^38^ cardiac glycosides (CGs, digoxin, digitoxin, ouabain and lanatoside C),^22^ oxaliplatin,^39^ bleomycin (BLM, an antitumour antibiotic glycopeptide),^40^ cyclophosphamide (CTX),^41^ and shikonin (SK),^42^ have been widely used in controlled studies as a criterion for the successful induction of ICD and the inhibition of tumour growth (Table 1). Furthermore, photodynamic therapy with hypericin has been verified to induce phox‐ER stress, which leads to the early induction of ecto‐CRT expression, active secretion of ATP and the passive release of heat shock proteins (HSPs), such as HSP70 and HSP90, in late apoptosis and strongly prevents tumour growth by inducing ICD in non‐immunized mice.^17^, ^43^, ^44^ Therefore, in recent years, an increasing number of researchers have devoted resources to discover novel inducers of ICD that could be successful and effective tools and contribute to indirect long‐term antitumour effects. Interestingly, regardless of the chemotherapeutic agents used, from the perspective of the induction of the pathway and its effect, ICD induction via physical therapy and combination therapy seems to have much more profound clinical and therapeutic implications than ICD induction via chemotherapy alone (Table 2).

**Table 1:** The classic immunogenic cell death (ICD) inducers and induction process

ICD inducers	Cell lines	Induction pathway	Induction effects
Bleomycin (BLM) (antitumour antibiotic glycopeptide)	MCA205, B16F10 cell lines, CT26cell line	ROS‐dependent ER stress; eIF2a phosphorylation; express hallmarks of DAMPs, release of ATP	ICD induced; exerts calreticulin‐, IFNγ‐ and CD8‐dependent antitumour effect^40^
Hypericin photodynamic therapy (Hyp‐PDT)	T24 cells; CT26 cell line; murine GL261 glioma cells	phox‐ER stress following overlapping molecular pathways consisting of the classical secretory pathway and phosphoinositide 3‐kinase (PI3K)‐mediated plasma; CRT relocation membrane/extracellular trafficking; release of HSPs such as HSP70 and HSP90	ICD induced; prevented the tumour growth in the non‐immunized mice^17^, ^43^, ^44^
Cyclophosphamide (CTX); in vitro active CTX derivative MAFO	RBL‐5 lymphoma; B16 melanoma cell lines	sCRT relocation; released substantial HMGB1	Induces an immunogenic type of apoptosis; promotes the engulfment by phagocytes^41^
Cardiac glycosides (CGs) digoxin (DIG), digitoxin (DIGT), ouabain and lanatoside C	Human osteosarcoma U2OS cells	Through the inhibitory effect on Na^+^, K^+^‐ATPase and consequent Ca^2+^ influx through the plasma membrane then induce CRT exposure, ATP release and HMGB1 loss, exposure of heat shock protein 90kD (Hsp90)	ICD induced; exert a positive impact on overall survival in cohorts of the breast, colorectal, head and neck and hepatocellular carcinoma in vivo and enhance the efficacy of non‐immunogenic anticancer therapies^22^
Shikonin	B16 melanoma cells	Activate both receptors‐ and mitochondria‐mediated apoptotic pathway; increase the expression of DAMPs and tumour‐associated antigens (TAAs)	ICD induced; activates dendritic cells to a high maturation status; enhances the priming of Th1/Th17 effector cells^42^
Anthracyclines (doxorubicin, idarubicin and mitoxantrone); all the chemical PP1/GADD34 inhibitors (tautomycin, calyculin A and salubrinal)	CT26 cells; B16F10A2/gp100 melanoma cells; DU145, OV90 cell lines; acute lymphoblastic leukaemia cells	Dephosphorylation of eIF2a; caspase activation; CRT relocation; express hallmarks of DAMPs	Induce immunogenic apoptosis and autophagic cell death; immunogenic dying tumour cells are efficiently phagocytosed by myeloid and plasmacytoid DCs stimulate the antitumour immune response; act as a strategy of immunogenic chemotherapy for the cure of established cancer^15^, ^37^, ^38^
Oxaliplatin	Balb/c mice with CT26 cells; RKO and HCT116 colon cancer cell lines	Stimulate the translocation of CRT from the endoplasmic reticulum to the cell surface CRT via the PERK/eIF2α/caspase 8/Bap31 axis; induce the release of HMGB1 from the nuclei	ICD induced; determines its therapeutic efficacy in CRC patients^39^

**Table 2:** The emerging ICD inducers and induction process

ICD inducers	Cell lines	Induction pathway	Induction effects
Near‐infrared photoimmunotherapy	A431 cells	Rapid and irreversible damage to cell membrane; cell swelling and bursting; Hsp70/90 and CRT relocation to the cell surface; ATP and HMGB1 release from cells into the medium	ICD selectively induction; maturation of immature DCs; elicited a host immune response against tumour^45^, ^46^
Oxygen‐boosted PDT of C@HPOC	4T1 cells; 4T1 mTNBC murine model	Increasing expressed critical DAMPs: ecto‐calreticulin (CRT), ATP and HMGB1	ICD‐induced; maturation of immature DCs; activation of T lymphocytes, natural killer cells (NK) and tumour‐draining lymph nodes (TDLNs); trigger ICD‐based antitumour immune responses^47^
Oncolytic peptides RT53 and LTX‐315	U2OS cell line	Trigger CRT exposure in a caspase‐ and eIF2α‐independent pathway; ATP and HMGB1 release from cells into the medium	ICD induced; infiltration of T cells increased; total cell killing and tumour growth blockade and regression^48^, ^49^, ^50^
LXR agonists T0901317	CT26 cells	Translocated CRT in eIF2α‐dependent; ATP and HMGB1 release from cells into the medium	Elicit tumour vaccine efficacy by inducing ICD^51^
Pt^II^ N‐heterocyclic carbene complex	CT‐26 and J774 cells	Trigger focused ROS‐mediated ER stress; express hallmarks of DAMPs: ecto‐calreticulin (CRT), ATP and HMGB1	ICD induced; exert immune‐modulating properties^52^
High hydrostatic pressure	Leukaemia, ovarian cancer and prostate cancer cell lines	Phosphorylation of eIF2a; activation of caspase‐3, ‐8 and ‐9; release of cytochrome c into the cytosol; expression of HSP70, HSP90 and CRT; extracellular ATP levels increased	ICD induced; DC phagocytosis up‐regulation of CD83, CD86 and HLA‐DR; release of interleukin IL‐6, IL‐12p70 and TNF‐a^53^
Oncolytic virus	Her2/neu TUBO‐derived tumours; prostate cancer cells	Increase the number of HER‐2–specific CD8^+^TILs secreting IFN‐γ; increased intratumoural infiltration of tumour antigen‐specific CD8^+^T cells	ICD induced; immunogenicity of the tumour‐associated antigens enhanced, breaking immunologic tolerance^54^, ^55^, ^56^
Engineered oncolytic vaccinia virus WR	MCA205 sarcoma cells; CT26 cells	Express hallmarks of DAMPs; release of ATP and CXCL10	ICD induced; the level of ICD was related to WR load and replication^57^
Nanoparticle‐encapsulated oxaliplatin	Pancreatic cancer cell line Panc‐1 and Pan02	Express more hallmarks of DAMPs	Enhanced ICD induction; induced a higher proportion of tumour‐infiltrating activated cytotoxic T lymphocytes; improved antitumour effects than the free ICD inducer^58^
ECT combine with ICOS activation	Lewis Lung Carcinoma (LLC) and CT26 cells	CRT membrane externalization increased; ATP and HMGB1 release from cells into the medium; ICOS promote the activation of T cells	ICD induced; tumour growth decreased in vivo; exert total tumour clearance with long‐term tumour‐specific immunological memory^59^, ^60^
Nanopulse stimulation	4T1‐Luc cells	Stimulates both caspase 3/7 activation; express three critical DAMPs: ecto‐calreticulin (CRT), ATP and HMGB1	ICD induced; trigger immune responses^61^, ^62^
RIG‐I‐like helicases	Panc02 and T110299 tumour cells	Expression of pro‐inflammatory cytokines type I IFN; up‐regulation of MHC‐I molecules and CD95 (Fas); CRT relocation to the cell surface; release of HMGB1 and HSP70	ICD induced; maturation of immature DCs; CD8a^+^ DCs efficiently engulf apoptotic tumour cells; induce efficient antitumour immunity in vivo^63^
ADC conjugated with PBD or tubulysin payloads	CT26 cells; mouse CT26 tumour model	Decreased the percentage of CD11c^+^MHCII^hi ^mature DC; increased per cent of F480^+^CD80^+^ macrophages	EphA2‐PBD is causing ICD in vivo and may contribute to the antitumour activity^64^

### 3.1 Nanotechnology serves to induce ICD

Nanotechnology is an excellent technology for application in cancer therapy. In recent years, nanoparticle encapsulation has been verified to improve the activity of chemotherapy drugs by controlling the drug dosage precisely to decrease the toxicity of substances used in pharmacology and in a physical modality; nanopulse stimulation (NPS) delivers ultrashort electrical pulses to tumour cells. However, all of these cancer therapy effects have been found to be related to the induction of ICD.

#### 3.1.1 Nanosized drug carriers and nanopulse stimulation

To confirm the enhanced permeability and retention (EPR) effect of nanocarriers,^65^, ^66^ Liu et al combined the traditional ICD chemical inducers mitoxantrone (MIT) and celastrol (CEL) at a ratio of 5:1 and then delivered this combination to a melanoma tumour site with a TME‐responsive nanoparticle (NP).^67^ After MIT and CEL were loaded on the TME‐responsive NPs and injected into the tumour, the effective amount of drug in the tumour was significantly higher with nanodelivered drugs than with that of free drugs. In addition, standard markers for drug‐induced ICD, CRT and HMGB1 were detected by fluorescence imaging and MIT and CEL also effectively induced apoptosis due to ICD. Furthermore, compared to other free ICD inducers, there was a strong recovery of DC functions in CD103^+^ DCs that were significantly increased by nanodelivered inducers. Ultimately, the study found strong synergy between MIT and CEL in inducing ICD and when nanodelivered, these factors could enhance long‐term immune surveillance by recruiting tumour‐specific memory CD4^+^ and CD8^+^ T cells. Importantly, Liu et al performed biosafety‐related toxicological pathology analyses to ensure that their combination of nano‐chemo‐immuno‐therapy targeting tumour growth and metastasis had low toxicity and high safety. Nanodelivered MIT and CEL strengthened the ICD effect, while nanoparticle‐encapsulated oxaliplatin (NP‐OXA) induced the release of more DAMPs and enhanced the immune responses of DCs and T lymphocytes more than OXA treatment alone in vitro.^58^ The proposed nanomedicine approach may be combined with ICD inducers to enhance antitumour effects and minimize the side effects of chemotherapeutic drugs.

#### 3.1.2 Nanopulse stimulation

Compared to drug‐induced ICD, nanopulse stimulation (NPS), which uses ultrashort electrical pulses in the nanosecond range, seems to be safer and less invasive. Nanosecond pulses penetrate all cells and organelles in the tumour at high speed and large amplitude,^68^ which allows internal calcium ions to rearrange.^69^ The calcium ions ‘escape’ from the ER and trigger ER stress, prompting the release of more ROS.^70^, ^71^ All these approaches are applied to cancer treatment. In several cancer cell lines, NPS first activates caspase 3/7 to induce cell apoptosis and ecto‐CRT is then increasingly expressed on the cell surface. Surprisingly, the percentage of ecto‐CRT‐expressing cells is NPS energy‐dependent and the ability of NPS to induce ecto‐CRT expression is comparable to that of anthracycline treatment. Further, the secretion of HMGB1 and ATP is observed after NPS treatment.^61^ In fact, NPS, which may be responsible for releasing DAMPs through ICD and triggering DC antigen presentation, can eliminate high percentages of primary 4T1 tumours (75‐100%) by inducing the immune response and activating adaptive immune memory.^72^ Thus, NPS is defined as a physical method that induces ER stress‐dependent ICD and its unique clinical utility is worthy of further in‐depth research.

### 3.2 Oncolytic virotherapy synergizes with the host immune system to induce ICD

In several clinical and preclinical studies, oncolytic virotherapy exerted the most effective antitumour results when potent viral oncolysis induced specific immune responses against tumour antigens.^73^, ^74^ Nevertheless, the induction of ICD in the context of oncolytic virotherapy is an essential factor that contributes to both oncolytic effects and immune responses.

Oncolytic viruses (OVs) were first recognized for their unusual cancer‐killing abilities; they directly kill cancerous tissues with almost no side effects, as they spare normal cells.^75^ Currently, the ability of OVs to break cancer immune tolerance and stimulate antitumour immunity by enhancing the induction of ICD has been tested in several cell lines and in prostate cancer.^54^ WR (VV_WR_/TK^‐^RR^‐^‐FCU1) is an engineered vaccinia virus. After injection of WR into MCA205 sarcoma cells in C57BL/6 mice, researchers found a trend of increasing calreticulin exposure, increased release of ATP and CXCL 10, which drove chemokine secretion that subsequently recruited T cells and presentation of tumour antigen to T cells by activated APCs.^57^ However, when OVs are combined with traditional ICD inducers, such as MIT or oxaliplatin, they potentiate antitumour effects and even break cancer immune tolerance. The typical features of ICD, such as calreticulin surface exposure, HMGB1 and ATP release and ER stress, are observed during OV‐mediated oncolysis of cancer cells; exposure of all these DAMPs significantly increases the number of HER‐2 specific CD8^+^ tumour‐infiltrating lymphocytes (TILs) secreting IFN‐γ, increasing intratumoural infiltration by neutrophils and tumour antigen‐specific CD8^+^ T cells.^55^, ^56^ Consequently, OVs, either as a monotherapy or in combination with other immunogenic chemotherapies, might lead to breakthroughs in cancer immunotherapy.

### 3.3 Advanced physical induction strategies for ICD

Chemical drug treatment has always been the main means of combating cancer development; however, the severe side effects of chemotherapy, such as pain and hair loss, have also added to patient suffering. In recent decades, many physical therapies, such as electrochemotherapy (ECT) in combination with inducible T cell costimulator (ICOS) activation, have been found to inhibit tumour growth, metastasis and angiogenesis.^59^, ^60^ Moreover, studies have also demonstrated that the induction of ICD is responsible for the long‐term antitumour response elicited by physiotherapy.

#### 3.3.1 Near‐infrared photoimmunotherapy

Near‐infrared photoimmunotherapy (NIR‐PIT) is a combination therapy that includes near‐infrared radiation and an antibody‐photosensitizer conjugate^76^; interestingly, the antibody acts as a ‘guide’ that directly locks onto cancer cells with overexpressed antigen on the plasma membrane, while the photoactivated silica‐phthalocyanine photosensitizer IRDye700DX (IR700) is localized to the target cells and attracts the NIR light. Once target cancer cells are exposed to NIR light, a series of selective cytotoxic effects will be triggered and eventually results in cell death.^76^, ^77^ Nakajima et al elucidated the possible cytotoxic mechanism that induces the cell swelling and ICD induced by NIR‐PIT.^45^ After NIR light irradiation, minute plasma membrane damage causes ions and molecules of specific sizes to enter cells; similarly, Ogawa et al found that NIR‐PIT‐induced damage caused water to flow into cells, which led to obvious rapid swelling.^46^ Thus, NIR‐PIT‐induced membrane damage is responsible for subsequent immunogenic signal exposure and release by dying cells. NIR‐PIT‐treated tumour cells express increasing levels of calreticulin, HSP70 and HSP90 on the plasma membrane and rapid discharge of HMGB1 and ATP has also been detected. In addition, NIR‐PIT‐induced ICD can promote the maturation of immature DCs, which contribute to long‐lasting antitumour immunity. Therefore, the results of the first NIR‐PIT clinical trial are worth following.

#### 3.3.2 Oxygen‐boosted photodynamic therapy (PDT) with C@HPOC

Photodynamic therapy (PDT) has been reported to have the ability to kill cancer cells through manipulating photosensitizers and oxygen to generate reactive ROS, which trigger phox‐ER stress and induce antitumour immunity to eliminate residual or metastatic tumours effectively and selectively.^17^, ^33^, ^44^ However, hypoxia in solid tumour environments is beneficial for promoting tumour metastasis, but the lack of oxygen is a severe factor that decreases the efficacy of PDT.^78^, ^79^ To relieve the limiting effects of hypoxia on PDT, Chen et al loaded Ce6 with a bioinspired hybrid protein oxygen nanocarrier (C@HPOC), which is presented by a protein hybridization approach.^47^ C@HPOC remarkably increased the oxygen content in the tumour via the tumour‐targeted codelivery of a photosensitizer and oxygen; thus, C@HPOC provided large amounts of 1O_2_ and improved the efficacy of PDT. As this oxygen‐boosted PDT approach using C@HPOC can self‐replenish oxygen, the antitumour effects of PDT are significantly improved. In 4T1 murine breast tumour cells, C@HPOC‐mediated PDT successfully enhanced ICD by inducing increased cell surface CRT exposure and HMGB1 and ATP secretion. Immediately, C@HPOC‐mediated PDT induced ICD‐promoted DC maturation, which eventually activated antitumour immunity, while sufficient oxygen generation relieved the immunosuppression in the TME, further promoting the infiltration of CD8^+^ T cells into tumours.^80^ Therefore, PDT treatment with C@HPOC is a promising strategy for inducing ICD and evoking systemic antitumour immunity.

#### 3.3.3 High hydrostatic pressure

Hydrostatic pressure (HP) is an important environmental parameter, while (high hydrostatic pressure) high hydrostatic pressure (HHP) has effects on biomolecules, cellular processes and viability, which are the basis of the antitumour effect of HHP. According to the pressure, HHP has been divided into ‘physiological HHP’ (<100 MPa) and ‘non‐physiological HHP’ (>100 MPa).^81^ Fucikova et al identified the potential of HHP to influence the type of cell death that occurs in human cancer cell lines (leukaemia, ovarian cancer and prostate cancer), as well as in primary lymphocytic leukaemia cells. This study showed that HHP treatment causes apoptosis and the expression of immunogenic molecules such as HSP70, HSP90 and CRT, on the cell surface and HHP‐treated cells also release HMGB1 from the nucleus and increase extracellular ATP levels. These characteristic ICD hallmarks then interact with the corresponding receptors on DCs, leading the DCs to acquire an immunostimulatory phenotype that activates antigen presentation and induces the activation of tumour antigen‐specific CD8^+^ and CD4^+^ T cells.^53^ Further investigations have shown that, the induction of ICD via HHP is mediated by ER stress; thus, HHP can be applied to cancer therapy as a reliable and potent inducer of ICD.

## 4 CONCLUSIONS AND FURTHER PERSPECTIVES

**Figure 3:**
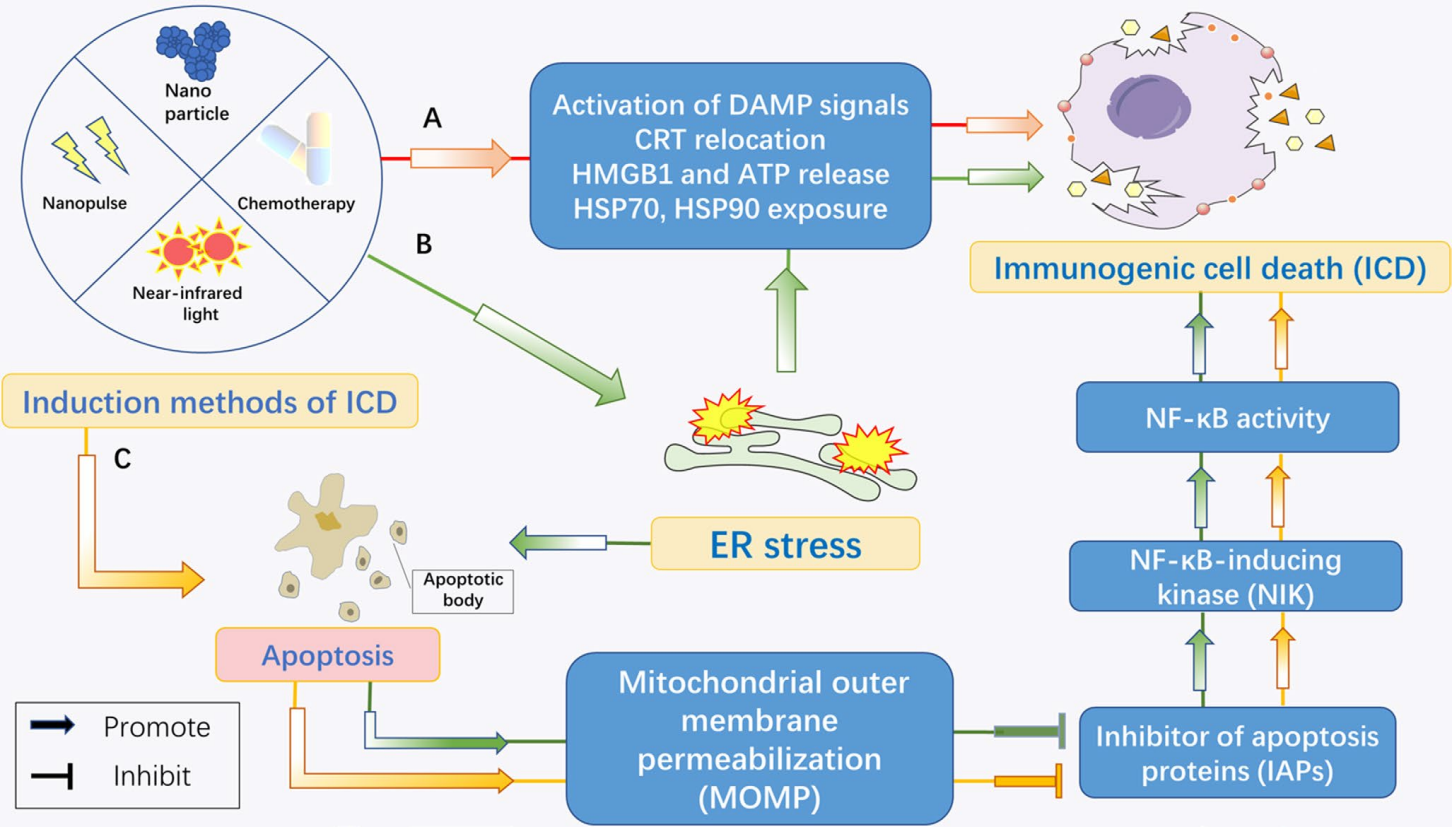
The induction pathway of immunogenic cell death (ICD). In the figure, there are three main pathways of ICD induction. Most ICD inducers are endoplasmic reticulum (ER) stress dependent, inducing the expression of damage‐associated molecular patterns (DAMPs) and triggering classic immunogenic cell death. Mitochondrial outer membrane permeabilization (MOMP) is another factor that stimulates NF‐κB activity and subsequently induces caspase‐independent cell death (CICD), which is also regarded as ICD

Since 2005, when the definition of ICD was first proposed,^37^ cancer treatment via host immunity has reached new heights. ICD makes dying cancer cells immunogenic and promotes DC maturation and IL‐1β production; in addition to activating immune cells, ICD induces antigen presentation by APCs, resulting in a long‐lasting antitumour response.^82^ The mechanisms of ICD induction have been clearly classified into two modalities.^83^ Type I ICD is induced through the collateral ER stress effect, indirectly inducing ICD‐associated danger signalling without triggering ROS production and ER stress. In contrast, Type II ICD induction selectively targets the ER, inducing ER stress‐dependent cell death, which is also immunogenic.^84^ However, further research has shown that the exposure and release of DAMPs are not the only characteristics of ICD. MOMP stimulates the activity of NF‐κB, which has recently been described as a vital immunogenic determinant of necroptotic cell death.^85^ To confirm the role of immunity in mediating the antitumourigenic effects, of CICD, Giampazolias et al performed MOMP on BALB/c mice (transfected with BCL‐2‐dependent control or APAF‐1‐knockdown CT26 cells). In this case, in tumours responding to CICD, analysis of tumour‐infiltrating T cells revealed increases in CD4^+ ^T helper cells and cytotoxic CD8^+^ T cells. Moreover, it has also been reported that IFNγ^+^ CD4^+ ^T helper cells and activated cytotoxic CD8^+^ cells (IFNγ^+^, GZMB^+^, IFNγ^+^/GZMB^+^) were increased in tumours responding to CICD, consistent with the activation of an antitumourigenic Th1 response.^19^ All these data indicated that for antitumourigenic effects of CICD, both NF‐κB activity and immunity are required and that CICD is an immunogenic form of cell death (Figure 3). Therefore, it is necessary to focus on ICD inducers or combination therapies that induce ICD.

In this review, we summarized the emerging methods of ICD induction, which are mostly combination therapies that use targeted drugs and medical technology to take advantage of the strengths of their components and compensate for the deficiencies of each component. These novel inducers elicit strong antitumour effects on cell lines and murine models via ICD induction; moreover, researchers have found that C@HPOC‐mediated PDT and NPS can both trigger ICD signals in the TME, subsequently activating DC maturation and causing T lymphocyte‐mediated antitumour immunity.^47^, ^72^ Importantly, ICD not only inhibits primary tumours but also exerts abscopal effects and drastically suppresses distant or metastatic tumours.^47^, ^62^ Based on the results of studies inducing ICD, killing cancer cells in a way that produces long‐term antitumour immunity similar to the immunity induced in natural antitumour immune responses is essential. Additionally, there are still some special ICD inducers that we want to introduce briefly. Liver X receptor (LXR) is known to be involved in cholesterol transport and immune response regulation^86^; thus, whether LXR agonists can induce ICD in human or murine colon cancer cells have aroused research interest. In LXR agonist‐treated Balb/c mice, CRT and HMGB1 expression levels were increased and the LXR agonists exhibited tumour vaccine effects by inducing ICD.^51^ Similarly, the oncolytic peptides RT53 and LTX‐315 also trigger the exposure of CRT and the release of HGMB1 and ATP as obligatory signals for ICD^48^, ^49^, ^50^; however, concerning the induction mechanism, the activation of ICD by LXR agonists is eIF2α‐dependent, as LXR agonists increase the expression of P‐eIF2α, while oncolytic peptides do not. In addition, RIG‐I‐like helicases (RLHs),^63^ the PtII N‐heterocyclic carbene complex (Pt‐NHC)^52^ and antibody‐drug conjugates (ADCs) conjugated with pyrrolobenzodiazepine dimer (PBD) or tubulysin payloads^64^ also display ICD‐inducing abilities and have immune‐modulatory properties.

Currently, monotherapies with chemical drugs do not meet the requirements for effective cancer treatment; they are efficient and eliminate tumours completely, but severe cytotoxicity and side effects make it difficult for patients to withstand these therapies. Thus, nanotechnology physiotherapy or OVs combined with ICD chemical inducers and physical therapy that induces ICD have emerged as novel cancer treatments. It is inspiring that ICD can now be an artificially induced, but the effects of novel ICD inducers in clinical trials and how to precisely control the dose, pulse energy or NIR light exposure time are not yet known. In conclusion, ICD induction is a promising area to explore, based on the known induction mechanisms and molecular mechanisms of cancer ICD, and more research is urgently needed to determine guidelines for the clinical application of emerging ICD inducers.

## CONFLICT OF INTEREST

The authors confirm that there are no conflicts of interests.

## AUTHOR CONTRIBUTIONS

JZ performed the literature research, drafted the manuscript and made the figure; GW and HW revised the manuscript and directed the review to be more focused, (JZ and GW contributed equally to this work); YC made the table; YH, ZC and HW gave the final approval for the article to be published.
